# FSH receptor binding inhibitor impacts K-Ras and c-Myc of ovarian cancer and signal pathway

**DOI:** 10.18632/oncotarget.25139

**Published:** 2018-04-27

**Authors:** Suocheng Wei, Xiaoyun Shen, Luju Lai, Haoqin Liang, Yingying Deng, Zhuandi Gong, Tuanjie Che

**Affiliations:** ^1^ College of Life Science and Engineering, Northwest Minzu University, Lanzhou, 730030, P. R. China; ^2^ Research Center of Animal Cell Engineering and Technology of Gansu Province, Northwest Minzu University, Lanzhou, 730030, P. R. China; ^3^ School of Karst Science, Guizhou Normal University, Guiyang, 550001, P. R. China; ^4^ School of Life Science and Engineering, Southwest University of Science and Technology, Mianyang, 621010, P. R. China; ^5^ Medicine College, Northwest Minzu University, Lanzhou, 730030, P. R. China; ^6^ Key Laboratory of Functional Genomic and Molecular Diagnosis of Gansu Province, Lanzhou, 730030, P. R. China

**Keywords:** FSH receptor binding inhibitor, ovarian cancer, oocyte, K-Ras, signal pathway

## Abstract

The present study aimed to investigate FSHreceptor binding inhibitor (FRBI) effects on relative factors (K-Ras, c-Myc and Vascular endothelial growth factor (VEGF)) to ovarian cancer, and expression levels of FSH receptor (FSHR) mRNAs and proteins in the cumulus-oocyte complex (COCs), to determine changes of protein kinase A (PKA) in sheep granulosa cells, further to elucidate signaling pathway of FRBI action. COCs were cultured *in vitro* for 24h under supplementation of varying concentrations of FRBI (0, 10, 20, 30 and 40μg/mL) or FSH (10IU/mL). Concentrations of K-Ras, c-Myc, VEGF, cAMP and FSH were detected in IVM media fluids, respectively. The results showed that the concentrations of c-Myc, K-Ras and FSH of FRBI groups were gradually reduced with the increase of FRBI doses. VEGF level of the FRBI-4 group was significantly greater than control group (CG). Expression levels FSHR mRNA and protein and PKA of FRBI-3 and FRBI-4 groups were less than that of CG or FSH group (P<0.05 or P<0.01). Inositol trisphosphate (IP3) concentrations of FRBI-3 and FRBI-4 groups were less than FSH group (P<0.05). FRBI administration doses had significant negative correlations to levels or concentrations of K-Ras, c-Myc, VEGF, FSHR mRNA and protein and PKA protein. K-Ras had significant positive correlations with FSHR mRNA and protein and PKA protein. In conclusion, FRBI could promote the production of VEGF of sheep COCs. Higher doses of FRBI (30 and 40μg/mL) suppressed the production of c-Myc and K-Ras, and declined FSH concentrations in the IVM medium fluid, and decreased the expressions of FSHR at the gene and protein levels, additionally attenuated expression of PKA protein in the granulosa cells.

## INTRODUCTION

Ovarian cancer (oophoroma) is a type of cancer that affects one or both ovaries and glands of the uterus. Epithelial ovarian cancer (EOC) is the most lethal female reproductive organ malignancy. The early diagnosis of EOC becomes a key factor in improving the survival rate of patients [1]. The cumulus and granulosa cells in the follicles support oocyte development. The previous studies reported that the retrieval of cumulus-oocyte complexes (COCs) from small antral follicles has been proposed as an attractive option for preserving female fertility in young cancer patients, particularly when controlled ovarian hyperstimulation is unfeasible or unsuitable [2]. *In vitro* maturation (IVM) of oocytes was a safe and feasible technique for attempting to preserve female fertility in the emergency [3]. The immature oocytes retrieved during the caesarean section were capable of IVM and could lead to live births after fertilization. Therefore, the immature oocyte collection in the luteal phase was a rescue option for female fertility preservation [4, 5].

Follicle stimulating hormone (FSH) is an important ovarian epithelial cell growth-promoting factor [6, 7]. FSH functions via binding to its cognate receptor (FSHR). Studies have indicated FSHR is present at a higher level in the ovarian cancers and gynecologic malignancies [1, 8]. FSHR overexpression may be associated with enhanced levels of potential oncogenic pathways and increased proliferation in EOC cells. Therefore, inhibition of FSHR overexpression may be beneficial to suppress the tumorigenesis and progression of EOC [1, 9].

FSH receptor binding inhibitor-8 (FRBI), an octapeptide non-steroidal low molecule, had been purified from human and sheep ovarian follicular fluid. This FRBI, as an FSH antagonist, not only blocked the binding of FSH to FSHR, but also altered FSH action at the receptor level [10]. FRBI can initiate the primary signaling cascades via the production of the cAMP, thereby regulating steroidogenesis. Our initial study revealed that the maturation rates of four FRBI-treated COCs gradually declined as FRBI concentrations increased from 0 (control group) to 40μg/mL in the IVM medium. Apoptosis rates of COCs were gradually increased as FRBI dose increased from 0 to 40μg/mL. The highest apoptosis rate was detected in the 40μg/mL FRBI-treated COCs. No nuclear atypia was found. Our results revealed supplement of FRBI into the IVM media could reduce the maturation rate, enhance the apoptosis rate, and decreased proliferation capacity of sheep COCs [11]. FRBI could suppress the mRNA and protein expression levels of FSHR and LHR in sheep cumulus-oocyte complex (COCs), additionally decreased FHS production and increased estradiol (E2) production of sheep COCs [11, 12]. Up to date, no reports have as yet documented about FRBI effects on the ovarian oncogenesis and genes related to ovarian cancerin humans and animals [9, 13, 14].

FRBI can initiate the primary signaling cascades via the production of the cAMP and inositol trisphosphate (IP3), thereby regulating steroidogenesis. Currently, whether FRBI treatment of oocytes influences this signal transduction remains unknown [15, 16]. The exact mechanism of FRBI actions needs still to be explored [14, 17].

Tumor biomarkers play an important role in the carcinogenesis of ovarian cancer and serve as tools in the diagnosis of the cancers. K-Ras and c-Myc are key oncogenes in the carcinogenesis of ovarian cancer. Expression rates of K-Ras protein in EOC patients were higher than those in benign ovarian tumor and normal control women. This revealed that K-Ras may participate in the process of occurrence and development of the EOC [18]. The c-Myc is a regulator gene that is closely associated with the cell proliferation and differentiation, apoptosis and tumorigenesis. The overexpressions of c-Myc proteins are likely to promote the process of malignant change and progression of tumor [8, 21]. Currently it is unknown whether FRBI impacts expression levels of K-Ras and c-Myc genes in follicles and ovarian epithelial cells [14, 18].

On the basis of our previous studies, the present work was undertaken to investigate FSH receptor binding inhibitor-8 (FRBI) effects upon the related factors (including K-Ras, c-Myc and VEGF, FSHR) to ovarian cancer, to evaluate the effect of FRBI on mRNA and protein levels of FSHR and ERβ in COCs, also to determine changes of protein kinase A (PKA) in sheep granulosa cells, further to elucidate signaling pathway of FRBI effects. We hypothesized FRBI impact these factors related to ovarian cancer, and expect to find a novel anti-cancer therapeutics.

## RESULTS

### Concentrations of K-Ras and c-Myc in IVM medium fluid

To evaluate the effect of FRBI on the production of K-Ras and c-Myc, ELISA assay was performed to detect the concentrations of K-Ras and c-Myc in IVM medium fluid.

#### K-Ras concentrations

**Figure 1:**
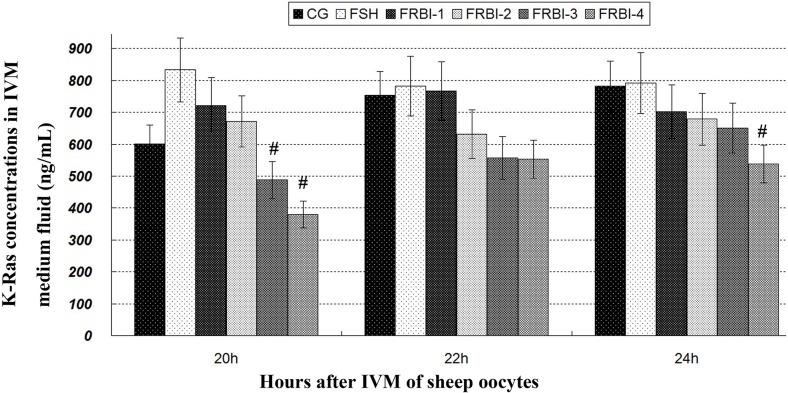
K-Ras contents in IVM medium fluid Addition of 0μg/mL FRBI into the IVM medium was used as the blank control group (CG). Addition of 10 IU/mL FSH into the IVM medium was taken as positive control (FSH group). Data was presented as means ± SEM. ^#^P<0.05 as compared to FSH group.

K-Ras concentration of FSH-treated group was slightly increased as compared to normal control (CG) after IVM of sheep COCs (Figure 1). But there was no significant difference. K-Ras concentrations of four FRBI-treated groups were gradually reduced, with a minimum reduction of the FRBI-4 group. K-Ras concentration of FRBI-4 groups was lower than FSH group (P<0.05) at 20 and 24 hours. The findings demonstrated that FRBI supplementation into the IVM media could decrease K-Ras production and levels in the IVM medium fluid.

#### C-Myc concentration

**Figure 2:**
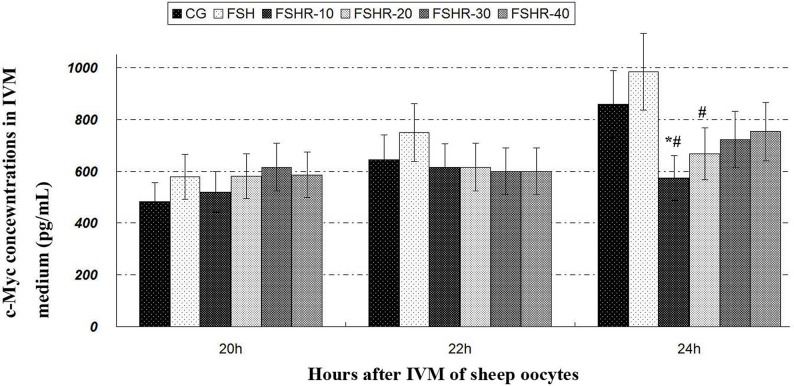
c-Myc contents in IVM medium fluid ^*^P<0.05 as compared to control group (CG); ^#^P<0.05 as compared to FSH group.

The c-Myc concentration of FSH-treated group was increased in comparison with CG (Figure 2). The c-Myc concentrations of FRBI-treated groups were decreased. The c-Myc concentrations of FRBI-1 and FRBI-2 were less than that CG or FSH group at 24h (P<0.05). The results indicated FRBI could suppress c-Myc production and decline level of c-Myc in the IVM medium fluid.

### Concentrations of VEGF and FSH in IVM medium fluid

VEGF concentration of FSH group was increased as compared to CG. VEGF concentrations of FRBI groups were gradually increased as the addition doses of FRBI increased. VEGF concentration of the FRBI-4 group was higher than that of CG (Table 1) at 24h after IVM (P<0.05).

**Table 1:** VEGF and FSH concentrations at 24h (Mean ± SEM)

Group	VEGF (pg/mL)	FSH (ng/mL)
CG	1713.4±162.2	54.7±6.2
FSH	2083.8±241.5	84.0±9.6
FRBI-1	1795.9±201.6	49.3±4.6^#^
FRBI-2	1896.1±167.2	42.1±5.0^#^
FRBI-3	2051.0±211.9	31.4±3.9^##^
FRBI-4	2175.5±223.7^*^	25.4±2.9^*##^

**Note:** Addition of 0 IU/mL FRBI into IVM medium was used as the blank control group (CG). Addition of 10 IU/mL FSH into the IVM medium was taken as positive control (FSH group).

^*^P<0.05 and ^**^P<0.01 as compared to control group (CG); ^#^P<0.05 as compared to FSH group. ^##^P<0.01 as compared to FSH group.

FSH concentration of in FSH-treated group was increased in comparison with CG (Table 1). FSH concentrations of FRBI treatment groups were dose-dependently decreased along with the increase FRBI doses from 10μg/mL to 40μg/mL. FSH concentrations of four FRBI groups were lower than that of the FSH group (P<0.05 or P<0.01).

These findings demonstrated that FRBI treatment could decrease production of FSH. A high dose FRBI (40μg/mL) accelerated VEGF production and raise the concentration of VEGF in the IVM medium fluid.

### Expression levels of FSHR mRNAs and proteins of sheep COCs

**Figure 3:**
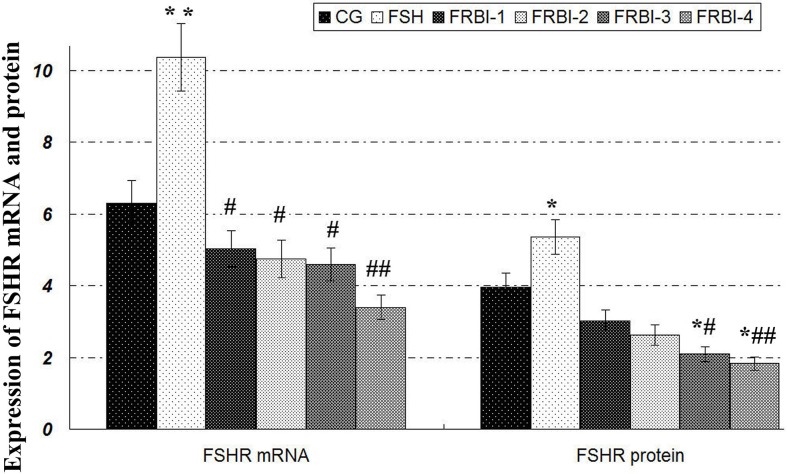
Expression levels of FSHR mRNA and protein Western blotting analysis showed that FSHR proteins were expressed in sheep COCs with β-actin as a reference gene. Expression levels of FSHR proteinof four FRBI-treated groups were gradually decreased as compared to CG and FSH group, with a minimum value of FRBI-4 group. ^*^P<0.05 as compared to control group (CG); ^#^P<0.05 and ^##^P<0.01 as compared to FSH group.

The expression levels of FSHR mRNAs and proteins of FSH group were enhanced as compared to CG (P<0.05) (Figure 3). Expression levels of FSHR mRNAs and protein in four FRBI-treated groups were gradually declined. FSHR mRNA levels of all FRBI groups were significantly less than FSH group (P<0.05 or P<0.01). Levels of FSHR proteins of FRBI-3 and FRBI-4 groups were significantly smaller than CG and FSH group (P<0.05 or P<0.01). These results demonstrated that FRBI treatment could dose-dependently suppress the FSHR levels of in sheep COCs at the gene and protein levels.

### Concentrations of IP3 and cAMP in IVM medium fluid

#### Inositol triphosphate (IP3) production

**Figure 4:**
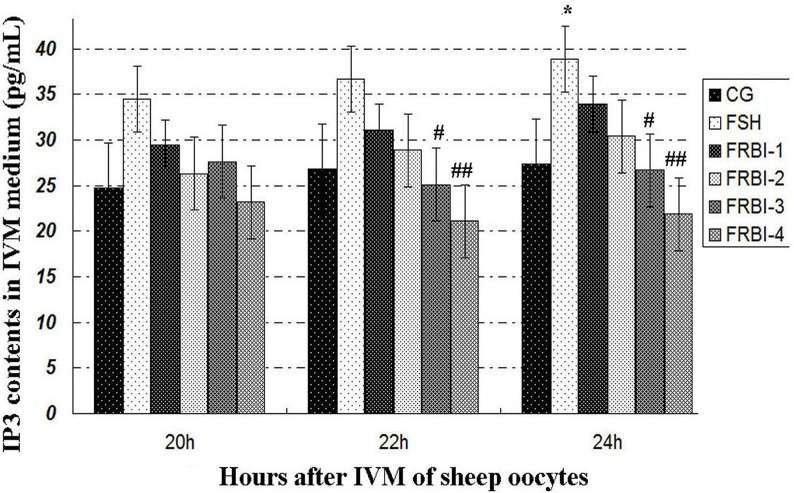
Inositol triphosphate (IP3) concentrations ^*^P<0.05 as compared to control group (CG); ^#^P<0.05 and ^##^ P<0.01 as compared to FSH group.

IP3 concentration of FSH group was increased in comparison with CG (Figure 4), with a significant increment at 24h (P<0.05 or P<0.01).

IP3 concentrations of FRBI-1 and FRBI-2 groups were also slightly raised when compared to CG. Contrarily, IP3 concentrations of FRBI-3 and FRBI-4 groups were less than that of the FSH group (P<0.05). The results demonstrated that lower doses FRBI (10-20μg/mL) slightly promoted IP3 production, but higher doses of FRBI (from 30 to 40μg/mL) reduced IP3 production.

#### The cAMP production

(Data omitted) The cAMP concentrations of FRBI-3 and FRBI-4 groups were slightly decreased when compared to CG and FSH group. However, there was no significant difference between all groups. The data indicated FRBI treatment had no obvious effect.

### PKA levels of sheep granulosa cells

**Figure 5:**
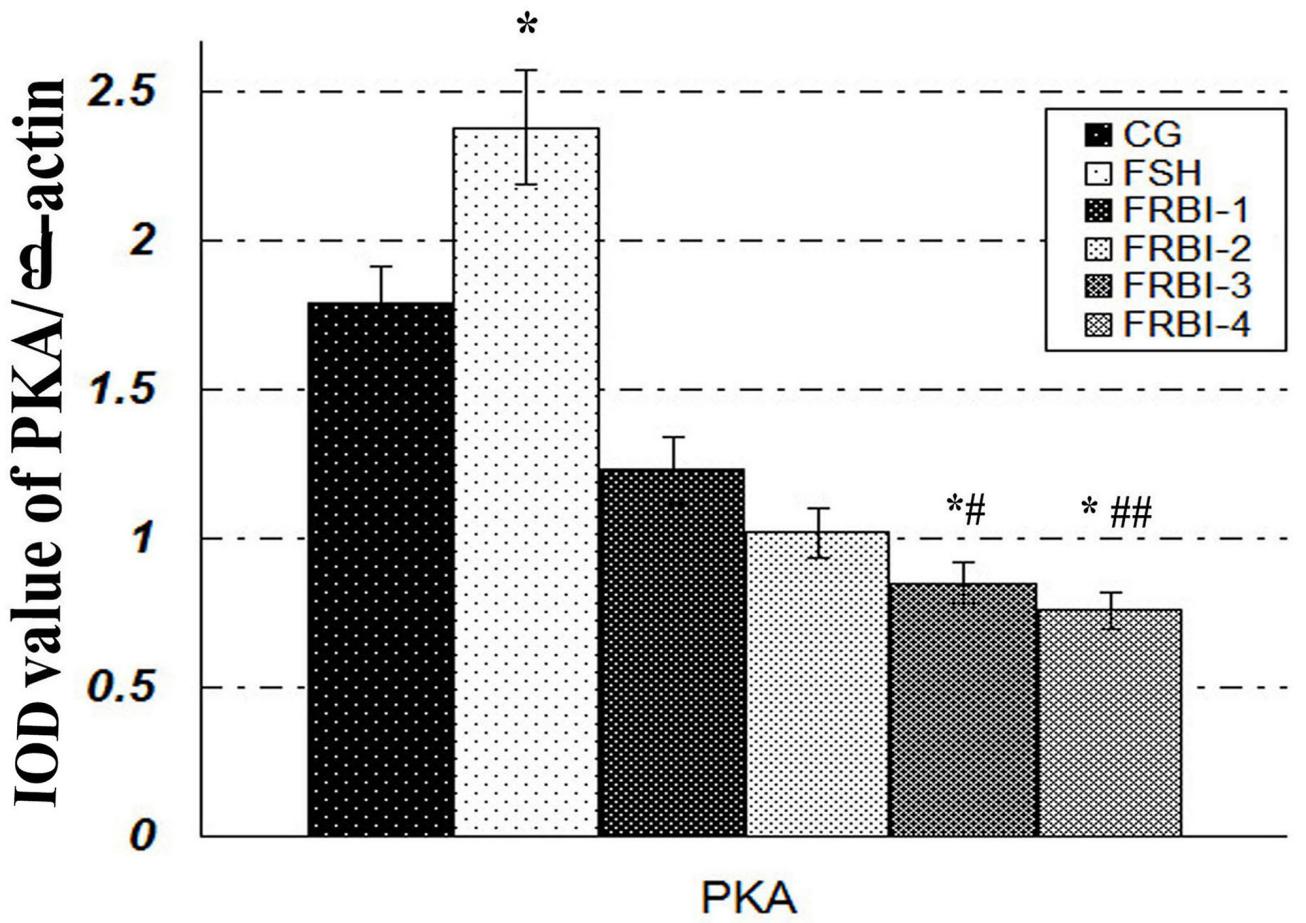
Effect of FRBI on protein kinase A (PKA) Sheep granulosa cells were stimulated with FRBI (from 0-40μg/mL) and FSH (10 IU/mL). Cells with 0μg/mL FRBI treatment were used as the blank control group (CG). Cells with 10IU/mL FSH treatment were served as the positive (FSH group). ^*^P<0.05 as compared to control group (CG); ^#^P<0.05 and ^##^ P<0.01 as compared to FSH group.

PKA was expressed in sheep granulosa cells (Figure 5). In comparison with CG, expression levels of four FRBI groups were gradually declined after FRBI simulation with the increase of FRBI supplementation into the IVM medium. PKA levels of FRBI-3 and FRBI-4 groups were lower than that of CG and FSH group (P<0.05 or P<0.01). The findings revealed that FRBI treatment could inhibit the expression of PKA in sheep granulosa cells. FSH treatment promoted expression of PKA.

### Pearson correlation analysis

As listed in Table 2, FRBI administered doses had significant negative correlations to levels or concentrations of K-Ras, c-Myc, VEGF, FSHR MRNA and protein and PKA protein. K-Ras had significant positive correlations with FSHR mRNA and protein and PKA protein. However, VEGF had significant negative correlations with FSHR mRNA and protein and PKA protein. There was a positive correlation between FSHR and K-Ras at the gene and protein levels.

**Table 2:** Pearsons correlation analysis between indexes

Items	Dose	c-Myc	K-Ras	cAMP	VEGF	FSHR m	FSHR p
c-Myc	-0.098						
K-Ras	-0.963^**^	0.164					
cAMP	-0.802	-0.11	0.784				
VEGF	0.902^*^	-0.315	-0.916^*^	-0.885^*^			
FSHRm	-0.950^*^	0.302	0.989^**^	0.721	-0.918^*^		
FSHR p	-0.977^**^	0.289	0.932^*^	0.749	-0.922^*^	0.948^*^	
PKA p	-0.975^**^	0.310	0.941^*^	0.742	-0.926^*^	0.959^**^	0.999^**^

Note: Casp-caspase-3; FSHR m-FSHR mRNA; FSHR p-FSHR protein; PKA p-PKA protein.

^*^ Correlation is significant at the 0.05 level (2-tailed). ^**^ Correlation is significant at the 0.01 level (2-tailed).

## DISCUSSION

K-Ras and c-Myc are main biomarkers and oncogenes in the carcinogenesis. The expression rate of K-Ras protein in EOC patients was higher than those in benign ovarian tumor and normal control women [18, 19]. K-Ras may participate in the process of occurrence and development of epithelial ovarian cancer [18]. C-myc positive expression rates were 53.9% in EOC tissues and 15.4% in normal ovarian tissues, respectively (*P*<0.001). C-myc can promote the progression, invasion and metastasis of EOC. Its detection may assist in early diagnosis of EOC [19]. The overexpression of c-Myc proteins probably promotes the process of malignant change and progression of tumor [20, 21]. Therefore, the Myc family is an excellent target for anti-cancer therapeutics [22, 23].

This study revealed that K-Ras and c-Myc concentrations in the IVM medium fluid were gradually reduced after COCs were treated with FRBI at different doses (from 10 to 40 μg/mL). FRBI could decrease K-Ras and c-Myc production and decline level of c-Myc in the IVM medium fluid. This prompted FRBI was likely to inhibit expression of K-Ras and c-Myc. FRBI blocked the carcinogenesis and progression of ovarian cancer. Currently, few document has reported similar outcomes, these findings have to be investigated in other animals and humans. Our study probably provided a new thinking for the prevention and treatment of humans and animal's cancers.

Angiogenesis is a crucial feature of EOC pathogenesis [22]. Vascular endothelial growth factor (VEGF) is the most important angiogenesis promoter [23, 24]. VEGF protein has been consistently associated with EOC progression [23, 25]. In this study, VEGF concentrations of four FRBI-treated groups were gradually increased as the addition doses of FRBI increased. VEGF concentration of the FRBI-4 group was higher than that of CG. The results indicated that a high dose FRBI (40μg/mL) treatment could enhance VEGF production and raise the concentration of VEGF in the IVM medium fluid.

FSH plays an important role in ovarian epithelial carcinogenesis. It stimulates the proliferation and invasion of ovarian cancer cells, increases the expression of VEGF and facilitates the neovascularisation [18]. But, other studies found no association between circulating FSH levels and ovarian cancer risk [26, 27]. Therefore, the actual efficacy of FSH carcinogenesis of ovarian cancers remains undetermined. In the present study, FSH concentrations of FRBI-treated groups were gradually declined, leading to significant reduction in comparison with CG and FSH groups. Our findings were consistent with the previous document [28]. However, the action mechanism has to be thoroughly explored.

The expressing level of FSHR increased from ovarian epithelial inclusions to benign epithelial ovarian cancer (EOC). FSHR overexpression may play a role in the early phase of EOC development [29]. However, the majority of studies are inconsistent and inconclusive results [8, 15, 30]. Our study showed the expression levels of FSHR mRNAs and proteins of FRBI-treated groups less than CG and FSH group. FRBI treatment could dose-dependently suppress FSHR levels of in the sheep COCs at the gene and protein levels. These possibly contributed to blocking the cancer progression and proliferation of tumor cells.

FSH exerts its role through signaling initiated by the protein kinase A and C (PKA and PKC) in granulosa cells [1]. PKA plays a central role in regulating signal transduction pathway and intracellular gene expression. One key signaling cascade regulated by FSH–FSHR interaction is the production of second messenger molecules (cAMP) and inositol trisphosphate (IP3). The cAMP and IP3 further lead to the activation of the downstream cascade of protein kinases. FRBI is able to fully inhibit FSH-induced cAMP production, thereby inhibiting steroidogenesis [30]. Up to date, whether FRBI treatment of oocytes influences this signal transduction remains unknown [15, 16]. The present investigation studied FRBI effects on PKA expression in ovine granulosa cells. This work revealed FRBI treatment reduced the production of the cAMP and IP3 of COCs, decreased PKA expression in granulosa cells. Our findings still need to be further investigated.

## MATERIALS AND METHODS

### Preparation of FSH receptor binding inhibitor (FRBI)

The FSH receptor binding inhibitor (FRBI) peptide of 99.9% purity was synthesized by Nanjing Peptide Biotech Co. Ltd., Nanjing, China (CAS: 163973-98-6) and was characterized before using it for experimental work. FRBI activity was determined by the radio receptor assay as reported earlier. To prepare FRBI solution (1.0mg/mL), 100mg FRBI was dissolved in 10% methyl sulfoxide (DMSO), the sterile normal saline was added into the above solution to a total volume of 100mL, the concentration of FRBI was 1000μg/mL. FRBI solution was kept at -20°C.

### Collections of sheep ovaries and classification of oocytes

Ovaries were collected between May to July of 2016 from 509 pre-puberty and noncyclic ewes (6-7 months old) immediately after their slaughter at the local shambles [31]. The collected ovaries were placed at 30-35°C in Dulbecco's phosphate-buffered saline (DPBS) (Sigma Co. Ltd, Beijing, China), and transported to the laboratory within 3 h. Use of these animals was approved by the Institutional Animal Ethics Committee of Northwest University for Nationalities, and all experiments were conducted according to the conventions of the Committee for the Purpose of Control and Supervision of Experiments on Animals in China.

Cumulus-oocyte complexes (COCs) were recovered from antral follicles (3.0-5.0mm in diameter) by gently cutting follicles with a scalpel. COCs were collected from each animal and pooled in groups. They were washed twice in Medium 199 (Sigma) supplemented with 0.68 mM l-glutamine (Sigma), 1 mM pyruvate, 20 mM HEPES (Sigma), 100 U/mL penicillin (Sigma), 100μg/mL streptomycin (Sigma), and 10% FBS (Invitrogen, Carlsbad, CA, USA). Only COCs with at least three layers of complete cumulus cells were considered suitable for IVM [27]. COCs taken from all animals were collected together in one instrument tray. A total of 1086 COCs was used only for subsequent experiments.

### *In vitro* maturation of COCs

*In vitro* maturation (IVM) of sheep oocytes was performed in accordance with the earlier methods [31, 32]. A microwell culture system was used in this study. Collected COCs were rinsed three times with extraction fluid, and pre-equilibrated for 3 h before IVM culture. At least 30 COCs were randomly taken from the instrument tray and placed in one culture well (Nunc Inc., Naperville, IL, USA) containing 600 μL maturation media covered with 300 μL mineral oil. The basal maturation medium for COCs IVM was supplemented with the different doses of FRBI (Nanjing Peptide Biotech Lmt., Nanjing, China) at 0, 10, 20, 30 or 40μg/mL and 10IU/mL FSH, respectively. They were allocated to control group (0μg/mL, CG), FRBI-1, FRBI-2, FRBI-3, FRBI-4 and FSH groups, respectively (Table 3). COCs were then left to complete their maturation at 38.5°C in an atmosphere of 5.0% carbon dioxide in humidified air for 24h. IVM medium fluid was collected at 20, 22 and 24h respectively [7]. The supernatant was separated and stored at -20°C for analysis.

**Table 3:** Experiment design of IVM of sheep oocytes

Group	Oocytes Numbers	Addition doses of FSH or FRBI into the IVM medium
CG	203	Neither FRBI nor FSH was added into the IVM medium. This is as a blank control.
FSH	174	10 IU/mL of FSH was added into the IVM medium, as a positive control.
FRBI-1	172	10 μg/mL of FRBI was added into the IVM medium.
FRBI-2	183	20 μg/mL of FRBI was added into the IVM medium.
FRBI-3	181	30 μg/mL of FRBI was added into the IVM medium.
FRBI-4	202	40 μg/mL of FRBI was added into the IVM medium.

Notes: FSH - Follicle stimulating hormone; FRBI - Follicle stimulating hormone binding Inhibitor; IVM - *in vitro* maturation.

### Determination of c-Myc and K-Ras concentrations in IVM medium fluid

Concentrations of c-Myc and K-Ras were determined utilizing c-Myc and K-Ras kits for sheep (ELISA), respectively, according to the manufacturer's instructions (Shanghai Bangyi, Biological Technology Co. Ltd, Shanghai, China). The samples were executed in triplicate. Analytical sensitivities were 0.10pg/mL (c-Myc) and 0.15ng/mL (K-Ras). The inter-assay CV was lower than 6%. The correlation coefficient of the standard curve was 0.9991.

### Detection of VEGF, FSH, IP3 and cAMP concentrations in IVM medium fluid

The concentrations of VEGF, FSH, IP3 and cAMP in the IVM medium fluid were detected using the especial ELISA kits for sheep, respectively, according to the manufacturer's instructions (Cusabio Biotech Co., Ltd. Wuhan, P. R. China; or Xinyu Biotech Co., Ltd., Shanghai, China). Analytical sensitivities were 0.40pg/mL, 0.10ng/mL, 0.10μg/L for IP3 and 0.20 nmol/mL, respectively, for VEGF, FSH, IP3 and cAMP. The intra-and inter-experimental coefficients of variation were lower than 2.71% and 6.72%. The correlation coefficient of the standard curve was more than 0.9960. All samples were tested in duplicate in the same assay. The detailed operation steps were presented in our initial research [33].

### Real time RT-PCR (qRT-PCR) of FSHR mRNAs

FSHR mRNA was determined using real time reverse-transcription polymerase chain reaction (qRT-PCR) and cloning techniques.

#### Primer design

The primers specific for FSHR (GenBank accession number: NM-001009289.1) were designed with Beacon Designer 7.0 software (Premier Biosoft International, Palo Alto, CA, USA) according to manufacturer's guidelines and Primer-BLAST at NCBI. Ovine GAPDH gene (GenBank accession number: HM-043737.1) was selected as the reference gene for normalizing expression levels of target genes. The sequences of the primers used in the qPCR were as follows: FSHR, forward 5′-TCTTTGCTTTTGCAGTTGCC-3′ and reverse, 5′-GCACAAGGAGGGACATAACATAG-3′; GAPDH, forward, 5′- CTTCAACAGCGACACTCACTCT-3′ and reverse, 5′- CCACCACCCTGTTGCTGTA-3′. Primers were synthesized by Beijing AoKeDingSheng Biotechnology Co. Ltd., China. The concentrations of the primers (100 nM, 200 nM, 300 nM and 500 nM) were evaluated, and formation of primer-dimers was evaluated using the melting curve analysis. Thus, only those concentrations of primers which showed dimmer-free reactions were used for the further analysis. Primers were synthesized by Beijing AoKeDingSheng Biotechnology Co. Ltd., China.

#### Total RNA extraction

After IVM under the different FSH concentrations in IVM media, total RNA of 30 COCs was extracted using the TRIzol reagent (Invitrogen, Beijing, China), according to the manufacturer's instructions, then reverse transcribed [31, 25]. The extraction was replicated three times with 30-40 COCs.

#### qRT-PCR detection of FSHR mRNAs

Expression level of FSHR mRNA was determined using qRT-PCR, respectively [33, 34]. The relative amount of FSHR mRNA was determined by the 2^–ΔΔCT^ method and normalized to an endogenous reference gene, GAPDH. Each sample was executed in triplicate, and each experiment was replicated three times with 30 COCs for each replicate.

### Western blotting of FSHR protein in COCs

To evaluate the expression levels of FSHR proteins in COCs of sheep, Western blotting was implemented referred to our previous reports [29, 30]. Rabbit anti-sheep FSHR polyclonal antibodies (Sigma, 1:200) and β-actin polyclonal antibody (1:1000) were diluted and incubated at 4°C overnight, followed by 1 h incubation with the appropriate secondary antibody (1:2000). Anti-β-actin mouse monoclonal antibody was diluted in 1:10000 for sample loading control. Blots were further developed using a chemiluminescence reagent (SuperSignal West Pico, Rockford, IL, USA). The integral optical density (IOD) of the scanned band images was obtained by using Quantity One software (Bio-Rad Company, Hercules, CA, USA). The relative concentration of FSHR protein was presented as the ratio between the gray values of FSHR divided by that of β-actin respectively. A negative control was performed without primary antibody. Assays were performed in triplicate.

### Western blotting of protein kinase A (PKA) in the granulosa cells

#### Preparation of sheep granulosa cells

The follicles of ovine ovaries were punctured with a needle to release the granulosa cells in the serum-free DMEM. The granulosa cells were washed and resuspended in serum-free DMEM. The cell number was determined by hemocytometer [28].

#### Protein extraction from granulosa cells

To evaluate the effect of FRBI on the signal transduction markers, the sheep granulosa cells were suspended in DMEM (2.5×106 cells/100 μL/tube) supplemented with different concentrations of FRBI (0, 10, 20, 30, 40 μg/mL) and FSH (10 IU/mL) at 37°C for 24h incubation on a shaking water bath. They were divided into the control group (CG), FRBI-1, FRBI-2, FRBI-3, FRBI-4 and FSH groups, respectively. Cells with 0 μg/mL FRBI served as the blank control (CG), and cells treated with 10 IU/mL FSH were taken as positive control (FSH group).

Then granulosa cells were centrifuged, the supernatant was removed, and the cell pellet was lysed with ice-cold lysis buffer (20 mM Tris, 150 mM NaCl, 2 mM EDTA, 0.1% SDS, 1% Triton X-100, 0.5% sodium deoxycholate, 10% glycerol and protease inhibitor cocktail) for 20 min on ice followed by centrifugation to extract the proteins. The supernatant was harvested and protein concentrations of the cell lysates were determined by micro-BCA protein assay kit (Thermo Fisher, Waltham, MA, USA) and the cell lysates were stored at -70°C till further use.

#### Western blotting of PKA

To assess the effect of FRBI onPKA expression of granulosa cells, Western blotting was performed as our earlier methods [7]. Briefly, 30μg proteins from each of the above cell lysates were resolved on a 12.5% SDS-PAGE for PKA detection. The specific anti-PKA antibody (Abcam Co. Ltd, USA) was used as primary antibody (1:200). The β-actin was used as the loading control in all the experiments. The experiments were conducted in triplicates.

### Pearson correlation analysis

Pearson correlation analysis was used to determine relationships between FRBI doses and ERβ mRNA and ERβ protein.

### Statistical data analyses

Statistical analysis was done using SPSS (version 21.0; Inc. Chicago, IL, USA). Data was presented as means ± SEM. All variables of five groups complied with the assumptions for a one-way analysis of variance (ANOVA). When significant differences were identified, supplementary Tukey's post hoc tests were performed to investigate pairwise differences. *P*<0.05 was considered to be significant.

## CONCLUSIONS

In the present study, we have, for the first time, found that FRBI treatment could accelerate production of VEGF of sheep COCs. Higher doses of FRBI (30 and 40μg/mL) suppressed the production of c-Myc and K-Ras, and declined FSH and IP3 concentrations in the IVM medium fluid, and decreased the expressions of FSHR at the gene and protein levels, additionally attenuated expression of PKA protein in the granulosa cells. These results contributed to our understanding the mechanisms of underlying FRBI modulation of biomarkers and oncogenes of ovarian cancer. Our findings provided a novel thought for finding the early prevention and therapy methods of ovarian cancer and other tumors of humans and animals.

## Abbreviations

cAMP: cyclic Adenosine monophosphate
CG: control group
COCs: cumulus-oocyte complexes
DPBS: Dulbecco's phosphate-buffered saline
EOC: epithelial ovarian cancer
FRBI: FSH receptor binding inhibitor
FSH: follicle-stimulating hormone
FSHR: follicle-stimulating hormone receptor
IP3: inositol trisphosphate
IVM: *in vitro* maturation
PKA: protein kinase A
VEGF: vascular endothelial growth factor
